# Leveraging Advances in Tuberculosis Diagnosis and Treatment to Address Nontuberculous Mycobacterial Disease

**DOI:** 10.3201/eid2203.151643

**Published:** 2016-03

**Authors:** Ravikiran M. Raju, Sagar M. Raju, Yanlin Zhao, Eric J. Rubin

**Affiliations:** Harvard School of Public Health, Boston, Massachusetts, USA (R.M. Raju, S.M. Raju, E.J. Rubin);; Chinese Center for Disease Control and Prevention, Beijing, China (Y. Zhao)

**Keywords:** bacteria, respiratory infections, tuberculosis, nontuberculous mycobacteria, Mycobacterium tuberculosis, Mtb, Mycobacterium leprae, NTM disease, M. abscessus, M. avium, M. ulcerans, antitubercular, antimycobacterial, macrolides, clarithromycin, oxazolidinones, bedaquiline, delamanid

## Abstract

The nontuberculous mycobacteria (NTM), defined as any mycobacterial pathogen other than *Mycobacterium tuberculosis* or *Mycobacterium leprae*, are a diverse group of pathogens that collectively cause a substantive but often unappreciated worldwide burden of illness. Although NTMs may cause illness similar to *M. tuberculosis*, these pathogens generally do not respond to classic tuberculosis (TB) drug regimens, resulting in misdiagnosis and poor treatment, particularly in resource-poor settings. Although a few high-quality epidemiologic surveys have been made on the topic, existing evidence suggests that NTM-associated disease is much more common than previously thought: more common than TB in the industrialized world and likely increasing in prevalence globally. Despite this evidence, these organisms remain markedly understudied, and few international grants support basic science and clinical research. Here we suggest that the considerable efforts in developing new treatments and diagnostics for TB can be harnessed in the fight against NTM-associated illnesses.

## Abstract 2

Recent advances in TB diagnosis and treatment must be considered in the basic scientific research of other mycobacterial diseases.

In recent years, major investments in basic research related to *Mycobaterium tuberculosis* have culminated in the large-scale rollout of the GeneXpert (Cepheid, Sunnyvale, CA, USA) diagnostic platform, the approval of bedaquiline for treatment of patients with drug-resistant tuberculosis (TB), and a deeper fundamental understanding of how the bacteria causes disease. These advancements stand in stark contrast to the poor understanding of the nontuberculous mycobacteria (NTMs). The NTMs are a group of organisms within the genus *Mycobacterium* (excluding *M. tuberculosis* and *M. leprae*) that cause a spectrum of diseases that include TB-like lung disease; localized infections of the lymphatic system, skin, soft tissue, or bone; and systemic disease (*1*). Previous studies have helped uncover NTM prevalence in industrialized countries in which differentiating between TB and NTM infections is much less challenging because of the availability of molecular techniques for detecting and identifying microorganisms. However, recent studies that have been done to examine the NTM burden of illness in industrialized settings have consistently uncovered an unexpectedly large prevalence (Figure).

**Figure:**
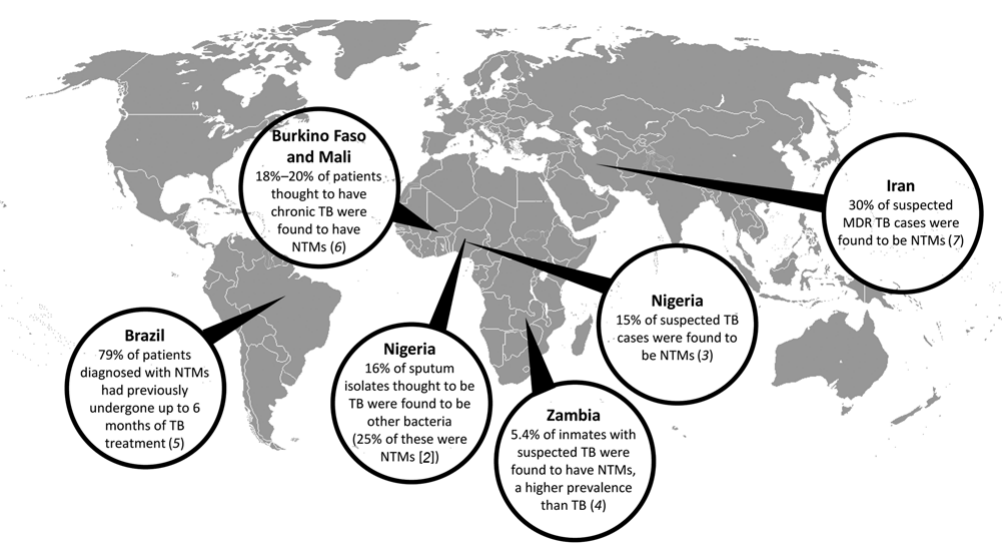
Summary of key studies of the epidemiology of nontuberculous mycobacteria (NTM) disease in countries populated by low- and middle-income residents. TB, tuberculosis; MDR TB, multidrug-resistant tuberculosis.

Major obstacles to adequately addressing NTM disease include the challenges of diagnosis and treatment as well as the lack of active research to understand the pathogenesis of these organisms. In each of these arenas, it is critical that we address gaps in the knowledge and capacity to deal with NTM-associated illness. By increasing funding to programs that seek to expand basic knowledge of NTMs and leveraging advancements in TB diagnostics and therapeutics, we can begin to form a deeper understanding of these pathogens and develop appropriate measures to address them. Here, we outline some of the challenges surrounding the diagnosis and treatment of NTMs and research of these organisms and propose avenues for how the road paved by the fight against TB can serve as a scaffold for advancing our understanding of these related, neglected pathogens.

## The Challenges of Diagnosis

NTMs share many characteristics with *M. tuberculosis* that make the bacteria difficult to differentiate in resource-poor settings. The standard method for diagnosing TB is through microscopic examination of sputum smears, but when this approach is used, NTMs appear identical to *M. tuberculosis*. Without molecular methods, which are unavailable in much of the developing world, these organisms are difficult to distinguish. Furthermore, in resource-limited settings, patients are often assumed to have *M. tuberculosis* infections because the clinical manifestations of many NTMs can mimic those of TB. In a study in Nigeria, Pokam et al. found that 16.5% of culture and sputum isolates thought to be *M. tuberculosis* were bacteria other than *M. tuberculosis* upon molecular typing; 25% of these misdiagnosed cases (4% overall) were found to be caused by NTMs (*2*). In another study in Nigeria, Aliyu et al. found that of 1,603 suspected TB cases, 15% were found to be NTM infections (*3*). Recent evidence has suggested that the rate of confusion between *M. tuberculosis* and NTMs may be even larger. Turnbull et al. discovered that inmates in a prison in Zambia who had symptoms of cough and an abnormal chest radiograph image showed an NTM prevalence of 5.4% compared with a 3.8% rate for *M. tuberculosis* (*4*). 

These findings have substantial implications for global health approaches to TB. Given that traditional treatments for *M. tuberculosis* infection are ineffective against most NTMs, the unexpectedly high rate of NTMs is likely a contributing factor to perceived TB treatment failure. In Brazil, a national mycobacterial referral center found that of 174 patients with pulmonary NTM, 79% had undergone TB treatment for up to 6 months before NTM infection was diagnosed (*5*). Studies conducted in Burkino Faso and Mali found that 18%–20% of patients suspected of having chronic TB were found to have NTM in their sputum (*6*). Similarly, a study in Iran showed that as many as 30% of suspected cases of multidrug-resistant TB were in fact NTMs, further suggesting the generalization of this phenomenon (*7*). Understanding the true prevalence of NTMs in the developing world is especially valuable considering that evidence suggests that NTM infection may interfere with the Bacille Calmette-Guérin vaccine, a widely used tool in preventing TB infections in the developing world (*8*).

These studies must be taken with some caution, as it is often difficult to distinguish whether the NTMs are a true source of infection or a contaminant in biological specimens or laboratory equipment. To account for this, the American Thoracic Society and the Infectious Disease Society of America guidelines for NTM diagnosis require isolation and growth of the pathogen on >2 separate occasions from the same patient to diagnose a pulmonary NTM infection (*9*). Because clinicians in most countries in the developing world often make diagnosis of TB on the basis of clinical symptoms, these guidelines may place a tremendous burden on laboratories in resource-poor settings. Any new diagnostic platform for the NTMs must account for the issues behind species differentiation and contamination and do so in a way that is feasible for application globally.

## The Challenges of Treatment

The difficulty of diagnosing NTMs and the frequent confusion of these pathogens with TB is compounded by the fact that standard TB treatments are often ineffective against NTM infections. Anti-TB medications produce a disappointing ≈50% response rate (*10*) in NTM-associated disease. As a result, misdiagnosis and mistreatment have huge implications on patient outcomes. Even within the NTM class, there is a substantial difference between the various species, which defies a one-size-fits-all treatment approach. This group encompasses pathogens with huge varieties of growth rates, host preferences, and inherent resistance to antibacterial drugs.

The introduction of macrolides, such as clarithromycin, for treatment for NTMs did improve cure rates for certain species, but in a retrospective study by Huang et al., many patients treated with these drugs for at least 12 months continued to have symptoms, and chronic illness was documented among patients who were successfully treated (*11*). Moreover, macrolide resistance is now well documented (*12*). The recommendation of multidrug regimens to counter such resistance is a logical next step, but often these regimens are minimally studied, and few if any have been investigated in a rigorous clinical trial. Therefore, while many clinicians rely on multidrug regimens for the treatment for NTM disease, the ideal combination of agents, duration of therapy, and true efficacy remain unvalidated and unknown.

## The Challenges of Current Research Paradigms

Fundamentally, poor understanding of the NTMs arises from a lack of investment. There have been few clinical studies of treatment for NTM-associated disease; most date from a time when advanced HIV infection was common in industrialized countries and opportunistic NTM infections were seen at an alarming frequency among HIV/AIDS patients. We conducted a search using the RePORTER tool (http://projectreporter.nih.gov/reporter.cfm) to find currently active grants from the US National Institutes of Health for this topic and found 228 grants related specifically to research on mycobacterial pathogens. Of these, only 5 (2.2%) were awarded to study specific aspects of the NTMs. These 5 grants cover a wide range of unmet needs, from understanding NTM susceptibility to novel drug discovery. However, this level of attention is clearly insufficient to address the many gaps that exist.

As prevalent as they are, many basic facts about the NTMs remain unknown. For example, it was largely thought that environmental exposure was the sole method of infection. However, a recent report that described whole genome sequencing as a molecular epidemiologic tool suggested that, in the context of cystic fibrosis patients, which is a population exceptionally susceptible to these pathogens, there may be a possibility of person-to-person transmission of *M. abscessus* (*13*). If true, control measures similar to those used for TB transmission might be effective, at least for highly susceptible persons. Although this study suggests that alternate modes of transmission may exist, it is still widely believed that environmental transmission is the major source of NTM infection, and numerous reservoirs such as household water sources have been identified (*14*). However, it can often be difficult to trace infections to a specific environmental source, which is a problem that is compounded by delayed and often incorrect diagnoses.

Additionally, it remains unclear why anti-TB medications are not effective treatment options. NTMs have complex cell walls, systems that modify both antibacterial drugs and their targets, and an extensive array of drug efflux pumps, but all these mechanisms also exist in *M. tuberculosis*. It may be that in NTMs, differing synergy of these and other mechanisms might conspire to produce a poor response to therapy. However, these differences must be studied to determine their significance. Such questions highlight the numerous areas in which our ability to address these pathogens would benefit from a better fundamental understanding.

## Addressing the Challenges: Leveraging Advancements in the TB Field

Although there are no simple solutions to the challenges of effectively addressing the NTMs, recent advancements in the TB field have potential for synergistic effects. One of the major breakthroughs in TB diagnostics over the past decade was the move toward using molecular methods such as the GeneXpert system. Although GeneXpert testing distinguishes fairly well between NTMs and *M. tuberculosis*, it does not distinguish within the broad category of NTMs, which is a necessary prerequisite to effective treatment. However, this advancement provides an opportunity to reconfigure molecular diagnostic platforms to include at least common NTM pathogens, providing a rapid and specific detection method. Even so, this method would not be a panacea. Because NTMs can colonize humans without causing disease and can contaminate biologic samples and laboratory equipment, simply finding the organism does not provide a definitive diagnosis. However, even raising the possibility can alert a clinician to consider the diagnosis of NTM-associated illness, limiting misdiagnosis and, as a result, incorrect treatment. These systems would also provide researchers with a tool to uncover the true burden of illness from NTMs.

Substantial efforts have been invested in anti-TB drug development. These have yielded 2 new approved antibiotics, bedaquiline and delamanid; several others are in clinical development. These drug discovery platforms could easily be transferred to screening for NTM-active compounds, and some of the new agents have already been shown to have activity against NTMs. For example, bedaquiline is more effective than currently existing antimycobacterial agents in treating *M. ulcerans* in a mouse model of infection and has shown promise as a salvage therapy for *M. avium* and *M. abscessus* (*15*,*16*). Moreover, new oxazolidinones, a class of drug effective against many NTMs, are currently being developed for TB and might prove clinically useful (*17*). Although many antitubercular compounds have poor activity against NTMs, new agents could serve as starting points that could be optimized; for example, bedaquiline derivatives have been found to have broad-spectrum activity (*18*).

While building on discoveries in the TB field, developing new interventions to diagnose, treat and prevent NTM-associated illness will likely require a better basic understanding of these organisms. Mycobacteria are extremely diverse; >150 species have been identified to date and vary in pathogenicity, virulence, mode of transmission, and antibiotic susceptibility. Here, too, the path paved by TB biologists could afford some insight into NTM physiology. The sequencing of the *M. tuberculosis* genome and the development of genetic tools to probe the bacterium’s physiology afforded novel insights into how this pathogen causes disease. Similarly, the sequencing of many NTM species, now being completed, could lead to novel discoveries that highlight why these organisms have been so difficult to treat in the past and why exactly they diverge from *M. tuberculosis* (*19*).

None of these measures will be taken without increased funding. The prevalence of NTMs not only in developing countries but also in the United States and many parts of Asia suggests that many public agencies should be willing to support NTM research. However, public funding is not the only avenue for increasing investment. Considering the prevalence and chronicity of these organisms in the industrialized world, a potentially substantial market of affected entities may exist. Resources from private interests and pharmaceutical companies could be leveraged to develop novel diagnostics and therapeutics if encouraged by advancements in the academic setting in our basic understanding of these pathogens.

The evidence that has been uncovered to date points to the NTMs as a source of substantial and growing burden of illness. NTM infections not only effect thousands of people, requiring lengthy and taxing treatments that are often not available in resource-poor settings; they also muddy the waters in the global fight against TB by draining resources resulting from misdiagnosis and mistreatment. Only through a concerted effort by researchers, clinicians, industry, and global health policy stakeholders to understand and address these issues can we respond to this large and neglected threat.

## References

[1] Holland SM. Nontuberculous mycobacteria. Am J Med Sci. 2001;321:49–55. 10.1097/00000441-200101000-00008 11202480

[2] Pokam BT, Asuquo AE. Acid-fast bacilli other than mycobacteria in tuberculosis patients receiving directly observed therapy short course in Cross River State, Nigeria. Tuberc Res Treat. 2012;2012: 301056. 10.1155/2012/301056 PMC3413975 22919477

[3] Aliyu G, El-Kamary SS, Abimiku A, Brown C, Tracy K, Hungerford L, Prevalence of non-tuberculous mycobacterial infections among tuberculosis suspects in Nigeria. Zhou D, editor. PLoS ONE. 2013;8:e63170. **PMID: 23671669** 10.1371/journal.pone.0063170 PMC3650061 23671669

[4] Turnbull E, Siyamabango M, Kaunda K, Chileshe C, Dunn I, Reid S, Clinical characterization of symptomatic patients with non-tuberculous mycobacteria in Zambian prisons [poster abstract]. Presented at: 44th^h^ Union World Conference on Lung Health; 2013 Oct 31–Nov 4; Paris, France.

[5] de Mello KGC, Mello FCQ, Borga L, Rolla V, Duarte RS, Sampaio EP, Clinical and therapeutic features of pulmonary nontuberculous mycobacterial disease, Brazil, 1993–2011. Emerg Infect Dis. 2013;19:393–9. 23745217 10.3201/eid1903.120735 PMC3647650

[6] Maiga M, Siddiqui S, Diallo S, Diarra B, Traoré B, Shea YR, Failure to recognize nontuberculous mycobacteria leads to misdiagnosis of chronic pulmonary tuberculosis. Hozbor DF, editor. PLoS ONE. 2012;7:e36902. **PMID: 22615839** 10.1371/journal.pone.0036902 PMC3353983 22615839

[7] Shahraki AH, Heidarieh P, Bostanabad SZ, Khosravi AD, Hashemzadeh M, Khandan S, “Multidrug-resistant tuberculosis” may be nontuberculous mycobacteria. Eur J Intern Med. 2015;26:279–84 . 10.1016/j.ejim.2015.03.001 25784643 PMC4414892

[8] Poyntz HC, Stylianou E, Griffiths KL, Marsay L, Checkley AM, McShane H. Non-tuberculous mycobacteria have diverse effects on BCG efficacy against *Mycobacterium tuberculosis.* Tuberculosis (Edinb). 2014;94:226–37. 10.1016/j.tube.2013.12.006 24572168 PMC4066954

[9] Griffith DE, Aksamit T, Brown-Elliott BA, Catanzaro A, Daley C, Gordin F, An official ATS/IDSA statement: diagnosis, treatment, and prevention of nontuberculous mycobacterial diseases. Am J Respir Crit Care Med. 2007;175:367–416. 10.1164/rccm.200604-571ST 17277290

[10] Wallace RJ, Brown BA, Griffith DE, Girard WM, Murphy DT, Onyi GO, Initial clarithromycin monotherapy for *Mycobacterium avium*-intracellulare complex lung disease. Am J Respir Crit Care Med. 1994;149:1335–41. 10.1164/ajrccm.149.5.8173775 8173775

[11] Huang JH, Kao PN, Adi V, Ruoss SJ. *Mycobacterium avium*–intracellulare pulmonary infection in HIV-negative patients without preexisting lung disease: diagnostic and management limitations. Chest. 1999;115:1033–40. 10.1378/chest.115.4.1033 10208205

[12] Nash KA, Inderlied CB. Genetic basis of macrolide resistance in *Mycobacterium avium* isolated from patients with disseminated disease. Antimicrob Agents Chemother. 1995;39:2625–30. 10.1128/AAC.39.12.2625 8592991 PMC163001

[13] Bryant JM, Grogono DM, Greaves D, Foweraker J, Roddick I, Inns T, Whole-genome sequencing to identify transmission of *Mycobacterium abscessus* between patients with cystic fibrosis: a retrospective cohort study. Lancet. 2013;381:1551–60. 10.1016/S0140-6736(13)60632-7 23541540 PMC3664974

[14] Thomson R, Tolson C, Carter R, Coulter C, Huygens F, Hargreaves M. Isolation of nontuberculous mycobacteria (NTM) from household water and shower aerosols in patients with pulmonary disease caused by NTM. J Clin Microbiol. 2013;51:3006–11. 10.1128/JCM.00899-13 23843489 PMC3754680

[15] Philley JV, Wallace RJ, Benwill JL, Taskar V, Brown-Elliott BA, Thakkar F, Preliminary results of bedaquiline as salvage therapy for patients with nontuberculous mycobacterial lung disease. Chest. 2015;148:499–506 and. 10.1378/chest.14-2764 25675393 PMC4694173

[16] Ji B, Lefrancois S, Robert J, Chauffour A, Truffot C, Jarlier V. In vitro and in vivo activities of rifampin, streptomycin, amikacin, moxifloxacin, R207910, linezolid, and PA-824 against *Mycobacterium ulcerans.* Antimicrob Agents Chemother. 2006;50:1921–6. 10.1128/AAC.00052-06 16723546 PMC1479135

[17] Vera-Cabrera L, Brown-Elliott BA, Wallace RJ, Ocampo-Candiani J, Welsh O, Choi SH, In vitro activities of the novel oxazolidinones DA-7867 and DA-7157 against rapidly and slowly growing mycobacteria. Antimicrob Agents Chemother. 2006;50:4027–9. 10.1128/AAC.00763-06 17015632 PMC1693997

[18] Balemans W, Vranckx L, Lounis N, Pop O, Guillemont J, Vergauwen K, Novel antibiotics targeting respiratory ATP synthesis in gram-positive pathogenic bacteria. Antimicrob Agents Chemother. 2012;56:4131–9. 10.1128/AAC.00273-12 22615276 PMC3421580

[19] Tateishi Y, Kitada S, Miki K, Maekura R, Ogura Y, Ozeki Y, Whole-genome sequence of the hypervirulent clinical strain *Mycobacterium intracellulare* M.i.198. J Bacteriol. 2012;194:6336 . 10.1128/JB.01439-12 23105072 PMC3486345

